# Nanoparticles for hematologic diseases detection and treatment

**DOI:** 10.15761/hmo.1000183

**Published:** 2019-06-28

**Authors:** Tania Limongi, Francesca Susa, Valentina Cauda

**Affiliations:** Department of Applied Science and Technology, Politecnico di Torino, Corso Duca degli Abruzzi 24, 10129 Turin, Italy

**Keywords:** nanomedicine, nanoparticles, theranostics, leukemia, lymphoma, blood diseases

## Abstract

Nanotechnology, as an interdisciplinary science, combines engineering, physics, material sciences, and chemistry with the biomedicine knowhow, trying the management of a wide range of diseases. Nanoparticle-based devices holding tumor imaging, targeting and therapy capabilities are formerly under study. Since conventional hematological therapies are sometimes defined by reduced selectivity, low therapeutic efficacy and many side effects, in this review we discuss the potential advantages of the NPs’ use in alternative/combined strategies. In the introduction the basic notion of nanomedicine and nanoparticles’ classification are described, while in the main text nanodiagnostics, nanotherapeutics and theranostics solutions coming out from the use of a wide-ranging NPs availability are listed and discussed.

## Nanomedicine and Nanoparticles

The word “nanotechnology” generally states the production of new tools with nanometric dimensions [1]. Most of the nanotechnology success relies on the possibility to adapt the structures and the design of a wide range of materials at the nanoscale to add or tune specific properties, thus significantly magnifying the materials science toolkit.

Nanotechnology is demonstrating its great potential in healthcare for theranostics, preventive applications and medical-designed novelties that are referred to as “nanomedicine” by the USA National Institutes of Health [2]. More in details, the nanomedicine research includes in vitro and in vivo medical diagnostics, nanopharmaceuticals and regenerative medicine applications [3–8].

Nanoparticles (NPs) represent one of the most successful solution that nanomedicine proposes to solve huge biomedical questions. With their size ranging from 1 to 100 nm, NPs are characterized by a considerable surface area-to-volume ratio. As described in [9] for diagnosis, therapy, and drug delivery applications, it is essential the obtainment of monodisperse NP preparations to avoid side effects coming out from aggregation phenomena. Starting from nanomaterials composition, tuning particles size, shape and functionalization states, it is possible enhance their in vitro and in vivo biodistribution, drug delivery and/or targeting capabilities.

Regarding their chemical composition, NPs can be distinguished into three main groups: carbon-based, organic and inorganic materials.

## Carbon-Based Nanoparticles

**Figure 1:**
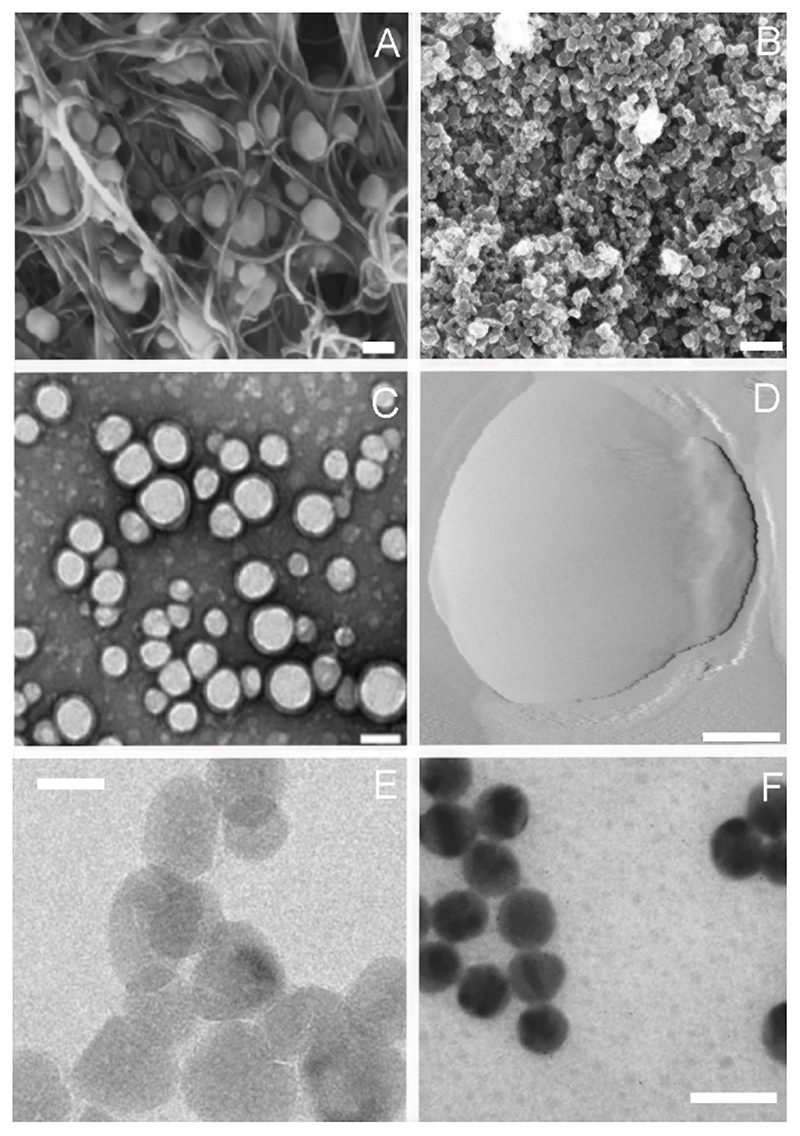
High resolution images of the main category of nanoparticles: carbon based (A,B), organic (C,D) and inorganic (E,F). (A) Scanning electron microscopy (SEM) of H5N2 AIV virions trapped inside the aligned nitrogen-containing multiwalled carbon nanotubes, scale bar 100 nm, adapted from [32]. (B) Field emission scanning electron microscopy images of carbon nanoparticles, scale bar 200 nm, adapted from [33]. (C) Transmission electron microscopy (TEM) image of PLGA nanoparticles, scale bar 100 nm, adapted from [34]. (D) Atomic force microscopy (AFM) image of a liposome, scale bar 50 nm, adapted from [35]. (E) TEM image of pristine ZnO nanoparticles, scale bar 20 nm, adapted from [31]. (F) TEM image of gold NPs synthesized by sodium citrate, scale bar 20 nm, adapted from [36]

The first group (Figures 1A and 1B) includes carbon nanotubes and fullerenes [10,11].

Fullerenes, water insoluble sphere containing 60 carbon atoms, represent the third allotophic form of carbon with respect to diamond and graphite. Fullerene derivatives have been successfully used for different diagnostics and therapeutics applications, many groups reported the use of these carbon allotropes for medical imaging and drug delivery purposes together with photodynamic, hyperthermia and acoustic wave assisted therapies [12,13].

Carbon Nano Tubes (CNTs) were classified into single-walled carbon nanotube (SWCNT) and multi-walled carbon nanotube (MWCNT) since they can be constituted by rolling up respectively one or more graphitic sheets. CNTs are applied in a wide range of biomedical applications as tissue engineering scaffold, biosensors and as labelling, imaging, drug delivery and therapeutic agents [14,15].

## Organic Nanoparticles

Concerning organic nanoparticles, we mainly refer to polymeric nanoparticles (PNPs), liposomes, and extracellular vesicles (EVs) (Figures 1C and 1D).

PNPs can be made from natural or synthetic polymers and thanks to their biocompatibility and biodegradability represent one of the most considered organic approaches for solve some nanomedicine challenges [16]. They can be produced by nanoprecipitation, dialysis methods, supercritical fluid technology, and two-step emulsification methods (emulsification-solvent diffusion, emulsification-solvent evaporation and emulsification–reverse salting-out). Their size and solubility can be tuned during the manufacturing process [17].

Liposomes are sphere-shaped vesicles made up of a lipid bilayer. They can be prepared starting from cholesterols, phospholipids, surfactants and proteins [18]. Liposomes can be synthetized by using different methods such as sonication, extrusion and the Mozafari method [19]. They can be considered as delivery systems able to carry both hydrophilic and hydrophobic drugs and molecules in their core, but are largely used for include also specific target biomolecules and other nanomaterials [20–22].

Regarding the extracellular vesicles (EVs) application in the biomedical contest, one of the most applied EVs categories is undoubtedly the nanosized exosomes (70–150 nm). They are released by all cell types, fine-tuning physiological and pathophysiological intercellular statements [23]. At present, exosome-like nanoparticles, naturally or synthetically obtained, represent some of the most capable, biocompatible, and therapeutic agents [9,22,24,25]. An European network of experts, the European Network on Microvesicles and Exosomes in Health and Disease (ME-HAD), reveals the open-ended capability of nanosized EVs for nanotheranostic investigations and applications [26].

## Inorganic Nanoparticles

The inorganic nanoparticles category includes Quantum Dots (QDs), Metallic (MNPs) and Metal Oxide (MONPs) Nanoparticles (Figures 1E and 1F).

QDs are semiconductor materials consisting of a core overcoated with a shell that is usually conjugated to peptides, proteins, polysaccharides and other biomolecules to prevent the leakage of the toxic-heavy metals and increase the overall NP stability in biological fluids. These kind of nanoparticles are the most used in bioimaging and biosensing strategies: gold quantum dots (GQDs), indium–phosphate (InP), cadmium–selenium (CdSe), indium–arsenate (InAs) cadmium– tellurium (CdTe) can be differently applied for real time cell tagging and cellular apoptosis recognition [20].

MNPs comprise magnetic and precious metals: MNPs such as Palladium (PdNPs), silver (AgNPs), gold (AuNPs), and copper (CuNPs) unveiled wide applicability as theranostics agents while magnetic ones exhibiting high stability in hypoxic tumor conditions and are successfully applied as contrast imaging and bio-sensing agents [27].

Biocompatible MONPs such as ceria (CeO_2_), mesoporous silica nanoparticles (MSNs), iron oxide (Fe_3_O_4_), zirconia (ZrO_2_), zinc oxide (ZnO) and titania (TiO_2_) show high chemical stability, antioxidant and catalytic actions that make them right for medical implants, drug delivery and bioimaging applications [22,28–31].

## Nanoparticles and Hematopathology

In order to assess data about the topic ‘blood nanoparticles’, in the April of 2019 we conducted a literature search, using the Thomson Reuters Web of Science research portal. Results showed 16,398 records that were visualized with the tree map style selecting 25 as number of results, sorting data by read count and setting the minimum record count to 285.

**Figure 2:**
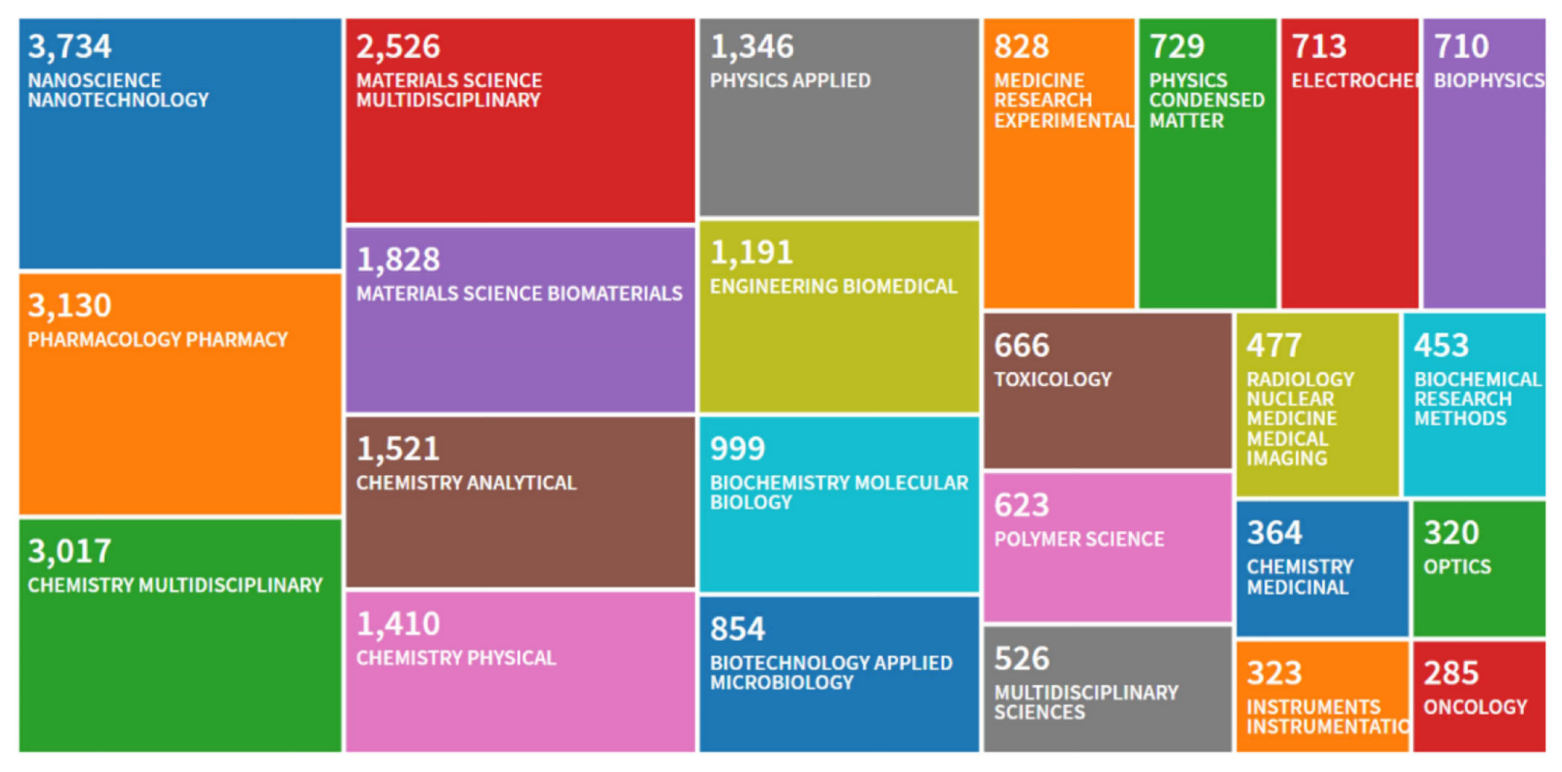
Thomson Reuters Web of Science research about the topic “blood nanoparticles”, made in April of 2019

The result (Figure 2) highlighted how wild and transversal is the literature that can relate to this type of research and it clearly shows how the scientific production is centred on nanotechological, pharmacological and chemical aspects.

In this paper we review how nanotechnology and more in details how nanoparticles could support and improve existing methods for early stages hematological diseases’ diagnosis and treatment, reducing side effects, relapses and costs.

Hematopathology studies the diseases related to blood, lymph nodes and bone marrow and, in this contest, nanoparticles-assisted nanomedicine can support researchers, physicians and clinicians providing complementary and/or alternative solutions to traditional diagnostic and therapeutic methods by providing effective and personalized solutions.

Considering that anemia, hemophilia, bleeding disorders and blood cancers as lymphoma, leukemia, and myeloma are just some of the many hematological pathologies, to emphasize the technological aspect of the discussion, in the next sections the three most broadly used NPs’ applications in nanomedicine will be presented: diagnostics, therapeutics and theranostics.

## Nanoparticle-Based diagnosis

Nanoscale diagnostic tools for early stage detection of cancer cells received a considerable attention in the recent years, in order to develop efficient methods able to isolate Circulating Tumor Cells (CTCs) from complex biological fluids.

In this contest, lymphoma cells present different non-glycosylated antigens on their surface. In particular, the CD20 antigen plays a critical role in the B lymphocytes activation and differentiation processes. The overexpression of this antigen on tumoral B cells made it a valid target to successfully isolate CTCs in blood and other human fluids. Many detection methods are based on active targeting moieties, such as ligands and monoclonal antibodies. In particular, anti-CD20, e.g. Rituximab, directed against CD20 antigen, associated with different types of nanoparticles, i.e. QDs or magnetic nanoparticles, aim to isolate lymphoma cells exploiting the high affinity between antigen and antibody.

Shariatifar, *et al.* proposed a new tool for the detection of non-Hodgkin lymphoma: Rituximab conjugated QDs bind specifically to tumor cells, allowing their detection through flow cytometry. Results display a higher sensitivity and specificity compared with immunohistochemistry, which is the current gold standard test [37].

QDs can be also conjugated with Sgc8c aptamer for an effective diagnosis of leukemia at the early stage by imaging tumor cells in vitro or in vivo [38].

Magnetic NPs characterized by having a high biocompatibility, stability, surface-to-volume ratio, binding-capacity and specificity were also successfully conjugated with anti-CD20 antibody to isolate lymphoma cells from biological fluids with an efficiency above the 95% [39]. At the same time, once functionalized with hyaluronic acids, they bound specifically to CD44 receptors, overexpressed in many types of leukemia and seized cancer cells from plasma samples. The subsequent analysis of the changes in mass loading, performed with a quartz crystal microbalance, detected the presence of tumor cells with high sensitivity, giving a feedback on cells’ condition and on drugs’ response [40].

CTC can also be detected with a customized device (biosensor), composed by a biological receptor and a physicochemical detector. They offer ease, quick and high sensitivity and specificity measurements of complex biological samples, in a very cost-effective process.

AuNPs have attracted attention as probes in biological detection for their biocompatibility, surface-to-volume ratio, ease of synthesis, surface functionalization and unique properties, among which the most important is their localized surface plasmon resonance (LSPR). AuNPs functionalized with thiolate oligonucleotides (Au-nanoprobes) can be used for the detection of bioanalytes, such as ions, proteins or target DNA at a lower cost comparing to traditional methods. The detection is carried out exploiting the LSPR, according to which their intense color changes together with the modification of the dielectric medium. Au-nanoprobes are efficiently applied to the direct detection of the molecular hallmark of chronic myeloid leukemia, BCR-ABL fusion transcripts, allowing the discrimination between the most frequent isoforms of this genetic abnormality, e13a2 and e14a2 [41]. Another sensor for the recognition of BCR-ABL is the BioCode Aunanobeacon which is constituted by AuNPs functionalized with hairpin shape strand DNAs with a fluorophore on the extremity. Due to their LSPR, AuNPs may act as dark quencher on the single strands’ fluorophores. In absence of target, the hairpin remains in the close conformation keeping the fluorophore near the AuNP, which quenches the fluorescence. If there is a bind between the hairpin and its target, instead, the hairpin opens and the distance from the AuNP allows the detection of the fluorescence [42]. Mazloum-Ardakani, *et al.* created a biosensor for acute lymphoblastic leukemia (ALL) early detection. By combining graphene sheets and an electrosynthesized conductive polymer, poly(cathecol), a high electrically conductive surface was realized and AuNPs were deposited on it in order to use their affinity with thiol-modified DNA to immobilize the target DNA [43].

AuNPs-based biosensors can be used also for the detection of specific factor of the coagulation cascade: thrombin-binding aptamer-conjugated AuNPs can detect thrombin [44,45], while peptide-functionalized AuNPs the factor XIII activity, exploiting the LSPR of NPs [46].

Graphene sheets, combined in different ways with aptamers, are also successfully used for the detection of blood cancer cells. Aptamers are artificial single-stranded oligonucleotides, having great affinity and selectivity with their targets, i.e. cells, proteins, drugs or small molecules, lower immunogenicity and toxicity, higher chemical and thermal stability and smaller dimension compared with antibodies. They can be used conjugated to nanoparticles for the detection of specific cancer cells, for example linked to carbon QDs coated with zinc oxide nanospheres [47] or to quantum dots coated with chitosan [48]. Graphene sheets are directly functionalized with Sgc8c and ATP aptamers [49] or with AuNPs conjugated with Sgc8c aptamer [50] to establish a bond with protein tyrosine kinase 7, overexpressed in T-cell of ALL. Sgc8c aptamer can be also used in combination with fluorescent mesoporous silica nanoparticles to identify leukemia cells through fluorescence microscopy [51] or with silver-enhanced AuNPs which selectively isolate and immobilize leukemia cells on a quartz crystal microbalance sensor surface to detect real-time changes in the resonant frequency [52].

A portable sensor for a rapid analysis and diagnosis of acute myeloid leukemia was developed by using Surface Enhanced Raman Scattering (SERS). Hollow core photonic crystal fibers are integrated with silver NPs in order to enhance the weak Raman signal of cancerous cells even at very low cells counts up to 300 cells/ml [53]. SERS AuNPs were also used to facilitate the detection of surface proteins such as CD45, CD19, CD20 of leukemia and lymphoma cells [54].

The diagnosis of multiple myeloma involves the detection of the Bence-Jones protein in serum. A complementary exam is the detection of the protein in urine, however the current analyses, e.g. immunoelectrophoresis, immunonephelometry and heat precipitation, are time consuming, have low sensitivity, and provide inaccurate results. Long, *et al.* proposed a new approach which is more rapid, sensitive and economical if compared to these current methods. They used macroporous ordered silica foams to enrich proteins in urine which are then analysed with matrix-assisted laser desorption/ionization time-of-flight mass spectrometry, to detect the presence of the Bence-Jones protein [55].

## Nanoparticle-based therapy

Many papers reported how MNPs’ and MONPs’ chemical-physical properties affect cancer cells viability. More in details silver NPs [56–59] and selenium NPs [60] are able to induce cells apoptosis on lymphoma cells in a dose dependent manner. Copper and cobalt oxide NPs display a selective cytotoxicity against hematological cancers cells through ROS generation or influencing p53 tumor suppressor gene activity [61,62].

Shahriari, *et al.* reported that L-asparaginase functionalized AuNPs are better internalized into leukemia T-cells than bare ones resulting more susceptible to localized hypertermia treatment [63].

Iron oxide NPs can enter B-cell lymphoma and multiple myeloma cells through phagocytosis and electrostatic interaction inducing cells autophagy and death. This induction of cells autophagy can be further non-invasively tuned by an external magnetic field [64] or by the addition of a chemotherapeutic agent, like bortezomib with gambogic acid [65].

Photodynamic therapy (PDT) is a non-invasive cancer treatment that is still under study but has shown great results. After the accumulation of a phosensitizer agent in the tumor, the diseased region is illuminated, usually with a laser source, and the photosensitizer transfer energy to molecular oxygen in order to generate ROS. ZnO NPs can act as photosensitizer [66] and their effects can be enhanced by the addition of chemotherapeutic agents, such as daunorubicin [67] or with other elements, such as manganese [68] to produce singlet oxygen which acts specifically against leukemic cells, without damaging healthy cells.

Metal NPs are promising nanocarriers for anticancer drugs, proteins or nucleic acids, thanks to their small size, biocompatibility and capacity to protect and deliver high payload of drugs selectively to the tumor by active or passive targeting methods.

AuNPs can deliver molecules with limited clearance, such siRNA [69], oligonucleotides that silence the BCR-ABL1 gene [70] or CpG and ovalbumin antigens that activate dT-cells reducing the tumor growth [71]. They can also be functionalized with anticancer drugs, such as 6-mercaptopurine [72], fludarabine phosphate [73], AS1411 [74] or dasatinib, a tyrosine kinase inhibitor, to reduce the effective dose [75].

Other chemotherapeutics can be loaded on different types of MNPs to improve their circulation time and the uptake by cancer cells, reducing side effects toxicity. For example, magnetic nanoparticles of magnetite can be coated with daunorubicin [76] or magnetite and silica with cytarabine [77], doxorubicin can be loaded on cadmium telluride QDs conjugated with PEG in order to also regulate the release in a pH-depedent manner [78]. Iron oxide NPs combined with paclitaxel and anti-ABCG2 monoclonal antibodies improve the therapeutic effect of the drug and reduce multiple myeloma progression [79].

Iron or iron-based NPs are successfully used for the treatment of a non-cancerous blood disease like anemia. It is commonly treated by oral administration of ferrous sulfate supplements, but only a small part is absorbed in the upper intestinal tract and the remaining reaches the low tract where reacts with hydrogen peroxide and superoxide producing free radicals and unfavourable effects. The reduction of the size of iron to the nanometric scale, increases its bioavailability and gastrointestinal absorption [80]. Further coating such as lipids [81,82], folic acid and chitosan [83] resulted to improve iron-based NPs stability and NPs inclusion in a bacteria, i.e. Lactobacillus fermentum, increased their efficiency [84]. In haemophilia treatment, Iron oxide NPs can be also coupled with factor VIIa for optimize its delivery [85].

MNPs can be applied also to coagulation disorders treatments: iron-derived NPs, such as magnetite, can be used as haemostatic agents. Magnetite matrix NPs, entrapping thrombin, are applied for a non-invasive treatment of internal bleeding: they are injected directly in the bloodstream, guided through an external magnetic field to the site of bleeding, where fibrinogen is injected to accelerate the coagulation process and the combination with thrombin-entrapped NPs stops the bleeding [86]. In contrast, some NPs can be used as anticoagulant agent: AuNPs coated with chitosan [87], the combination of AuNPs with thrombin binding aptamer produces nanoconstructs that interact with thrombin, inhibiting its activity, in a photo-controllable manner [88,89]. Zinc oxide NPs reduce the amount of thrombin and coagulation factors and prolong the time of thromboplastin and prothrombin [89]. Silver NPs, bare [90,91] or coated with chitosan [92], shown a high thrombolitic potential.

MSNs, entrapping the drug inside their pores efficiently deliver chemotherapeutics to cancer cells. Daunorubicin was encapsulated in mesoporous silica NPs functionalized with the B220 antibody to actively and selectively target acute leukemia cells [93]. Once encapsulated inside the pore, drugs have to be retained in the pore through stimuli-responsive coating that can act as a sealant and under specific condition slowly release the payloads. For example, a peptide responsive to the bind with a specific receptor overexpressed by tumors [94], pH-responsive PEG telomerase responsive oligonucleotide sequences [95], biotin-avidin and pepsin enzyme cap [96] or adenosine triphosphate and calcium carbonate [97] seal the pores and MSNs release their cargoes only in presence of specific condition typical of the different types of tumors, causing enhanced apoptosis and higher drug’s uptake. MSNs pores can be also sealed with lipid membranes creating a nanoconstruct called “protocell” [98] and the surface can be functionalized in different ways, for example by PEG binding, or targeting ligands [99].

MSNs can be employed as haemoglobin-based oxygen: exploiting the affinity between haemoglobin and MSNs, haemoglobin can be loaded inside the pores and replace the function of defective erythrocytes delivering oxygen to cells and tissues [100]. To control the release of haemoglobin MSNs can be coated with liposomes [101] while, for the controlled release of anticoagulant drugs, MSNs can be loaded with heparin. Once loaded with thrombin-specific cleavage sites and capped with a thrombin-sensitive peptide [102], MSNs can release the drugs to slow the coagulation cascade or if conjugated with thrombin binding aptamer and coated with a streptavidin cap, can be used for a reversible inhibition of thrombin activity toward fibrinogen [103]. MSNs were also covalently coated with heparin showing prominent anti-thrombogenic effects in whole blood from patient donors [104].

Among the many application of polymeric NPs in therapeutics, Poly(lactic-co-glycolic) acid (PLGA), as biodegradable polymer approved by FDA, is recognised as a valid drug delivery vehicle.

Curcumin, a natural hydrophobic yellow pigment with anticancer and anti-inflammatory properties, has low aqueous solubility and bioavailability so its drug delivery capability is enhanced by PLGA NPs incapsulation [105,106]. The same solution is used to deliver the antisense peptide nucleic acid that inhibits miR-155 expression to lymphoma cells: the withdrawal of this microRNA results in a rapid regression of the disease [107].

Barasertib or AZD2811 is a potent and selective Aurora B kinase inhibitor that has a pivotal role in inducing the chromosomes alignment during mitosis and controlling the cytokinesis, thus its inhibition induces polyploidization and cell death. The drug is encapsulated in polymeric nanoparticles called Accurine, composed of poly-D,L-lactide and poly(ethylen glycol). This drug delivery system allows a scheduling of weekly (or longer periods) administrations, thanks to the high dose delivered to the tumor that increases the durability of the response and decreases the toxic side effects [108,109].

To overcome some of the limitations of standard anticancer drugs, they can be encapsulated inside biodegradable polymeric NPs which guarantees the delivery of the drug to the target site and a continuous and controlled release. Chlorambucil and hydroxychloroquine [110–112] or doxorubicin [113] are loaded inside biodegradable polymeric NPs and are functionalized with anti-CD20 or anti-CD19 antibodies to specifically target different neoplastic B-cells. Idarubicin, an anthracycline antibiotic approved by FDA analogue to daunorubicin, can be encapsulated in methoxy poly(ethylene glycol)-b-poly(l-lactide-co-glycolide) nanoparticles [114].

A special reference needs to be made to nanogels that are colloidal hydrogel particles composed by three-dimensional crosslinked hydrophilic polymers networks. They are used for the delivery of hydrophilic large molecules, with the aim to protect drugs from degradation, and their surface can be functionalized to improve the blood circulation and the cellular uptake. Their drug delivery properties are based on the hydrogels’ capacity of swelling in aqueous environment instead of dissolving. Nanogels of linear thiolated poly(glycidol), modified with peptides sensitive to redox environment, loaded with the tumor suppressor miR-34a and a trans activator for transcription provide an effective tool for the treatment of multiple myeloma [115]. Other kinds of nanogels insert methotrexate in chitosan nanoparticles for intranasal administration in a central nervous system lymphoma [116].

Biodegradable polymers such as PCL or PLGA can be loaded with heparin [117], while chitosan show an intrinsic anticoagulant activity and can be combined with other polysaccharides, fucoidan and chondroitin sulfate, to produce NPs for the control of the coagulation cascade [118]. Chitosan NPs can be also loaded with factor VIII-encoding DNA and orally administered as an unconventional hemophilia A handling [119,120].

Two outstanding applications of PLGA nanoparticles have been studied for immune thrombocytopenic purpura and thalassemia, respectively. In the first case, a NP core of PLGA was covered by a platelet membranes coating, which presents all the typical platelet proteins able to partially neutralized the effects of anti-platelet antibodies and minimize the disease burden [121]. Thalassemia can be early detected during pregnancy, thus it is possible to administer intravenous or intra-amniotic PLGA NPs encapsulating peptide nucleic acid and donor DNA to correct the mutation of β-globin gene of the foetus [122,123].

There are several chemotherapeutic or anti-inflammatory drugs encapsulated in lipids and this practice has been proven to be effective enhancing the local concentration in inflamed tissues, reducing the exposure of other organs and also protecting the drug from degradation.

For instance, short interfering RNAs (siRNA) [124,125] or antisense oligonucleotides against Bcl-2 [126] can silence proliferation or antiapoptotic genes overexpressed in many tumors and, in combination with a lower dose of chemotherapeutic, can induce the remission of cancer. To prolong their circulation time, they can be encapsulated in liposomes and, to improve their target ability, functionalized with anti-CD20 antibodies.

Glucocorticoids are employed in clinic for the treatment of a variety of inflammatory driven malignancies, including multiple myeloma, eventually in combination with other chemotherapeutic drugs. However, they produce severe side effects like systemic immunosuppression, osteoporosis, hypertension and others, they have also a rapid clearance and a request of frequent administrations.

The therapeutic efficacy of dexamethasone is improved by liposomal encapsulation [127]. Some chemotherapeutic drugs, such as curcumin and doxorubicin [128], tamibarotene [129], carfilzomib and doxorubicin [130], gemcitabine [131], paclitaxel, docetaxel, SN38, etoposide, hydroxytamoxifen, miltefosine, or a ferrocenyl complex and decitabine [132], methotrexate [133], arsenic troxide [134], lead to a high complete remission of blood malignancies, but they cause also cardiac and other organs dysfunctions, protein inhibition and interaction with many biological reactions, so their use is limited for human treatment because of their toxic comorbidities. To specifically deliver these drugs to cancer cells, avoiding side effects, they can be encapsulated in liposomes. A special case of chemotherapeutic into liposomes is the ex-vivo modification of T-cells to carry SN-38-loaded lipid nanocapsule to lymphoma cells, taking the advantage of the tissue-homing ability of lymphocytes [135].

Liposomes can encapsulate drugs for different medical purposes: thrombin inhibitors, able to exert an anticoagulant activity in case of arterial acute thrombosis [136], mRNA to encode erythropoietin, factor IX protein [137] and anti-factor VIII antibodies [138,139] for haemophilia treatment and Tmprss6 siRNA for thalassemia cure [140].

Solid lipid NPs (SLNPs) are nanocarriers used as an alternative to polymeric NPs since they present a lipid core, which encapsulates lipophilic drugs controlling their release by increasing their solubility, bioavailability and pharmacokinetic profile in case of natural-derived anticancer drugs, such as AP9-cd [141] and curcumin [142]. SLNPs can reduce the toxicity of many chemotherapeutic drugs: hydroxychloroquine can be encapsulated in anti-CD20 functionalized PEG-PLGA NPs [143], daunorubicin and tetrandrine in PEG-PLL-PLGA NPs [144], parthenolide in PEG-PLA and silicon, bendamustine in PEG-PLGA [145], doxorubicin in PEG-PCL/Pluronic 105 micelles [146], paclitaxel in transferrin decorated SLNPs [147] and vincristine both in polyphenolic bioflavonoids [148] or folic acid decorated SLNPs [149].

## Nanoparticle-based theranostics

Various NPs act as multifunctional nanotools and can be used both for the detection and the treatment of such haematological cancers. Referring to AuNPs, their diagnostic capability is carried out exploiting their high absorption and scattering of light, while the high surface-to-volume ratio, afford their use as nanocarriers for drugs. AuNPs, with or without further funzionalization [150], sustain cancer diagnosis and the delivery of drugs such as the Fms-like tyrosin kinase inhibitors (midostaurin, sorafenib, lestaurtinib and quizartinib) [151–153].

Transferrin (Tf)-luminescent blue copper nanoclusters are coupled with doxorubicin for theranostics applications. When NPs are internalized in TfR overexpressed cells, it is possible to simultaneously detect the blue emission of transferrin into the cytoplasm and, the gradual release of doxorubicin in the nucleus through Förster Resonance Energy Transfer (FRET) [154].

Lanthanide-doped nanoparticles can be employed as bioimaging tool for their photoluminescent properties [155] and loaded with therapeutic cargoes and targeting molecules for a therapeutic use against acute myeloid leukemia cells [156].

Also liposomes can be engineered for theranostics application: they can be loaded with superparamagnetic iron oxide NPs (SPIONs) and detected by magnetic resonance or positron emission tomography for tracking and treatment monitoring. SPIONs are covered with liposomes and then functionalized with Rituximab for increase their targeting ability and, coated with tween80 to increase their circulation time and their penetration across the blood brain barrier (BBB) to reach also central nervous system lymphoma [157].

Core-shell chitosan-hyaluronic acid-NPs decorated with peptide pA20-36 were used to specifically target B-cell lymphoma and induce cells death in a caspase-dependent manner while fluorescent tracer and a paramagnetic agent allowed NPs internalization imaging [158].

Also Calcium phosphosilicate NPs can be engineered, once loaded with indocyanine green and functionalized with CD96 and CD117 antibodies, they can be endocytosed by leukemic cells, allowing the detection of the disease and the application of the photodynamic therapy [159].

## Conclusion

To conclude here we have reviewed the broad panorama of nanoparticles, which represent one of the most useful alternative solution to manage blood diseases providing innovative non-invasive approaches for diagnosis and treatment.

Although a large number of NPs-containing drugs have already received FDA approval (Table 1) or are at present involved in studies or clinical trials (Tables 2 and 3), NPs healthcare use requires improved chemical-physical characterization, better definition of their potential toxicity concerns and more detailed regulatory guidelines.

However, there is no doubt that the rapid progress of the engineering of new materials and the implementation of new methods in the nanotechnology field will lead to the design and standardization of alternatives therapies specific to each patient and disease. Nanomedicine, using original and multi-faceted instruments as the NPs are, can offer the precise targeting and therapeutics tools that researchers and physicians need, to make the diagnosis and treatment techniques that they already have at their disposal even more effective and competitive.

## Figures and Tables

**Table 1:** Drugs already approved by FDA for hematological disease treatment

Name	Composition		Disease	Year of approval	References
	NPs	Active substances			
CosmoFer/INFeD/Ferrisat	Iron dextrane colloid		Iron deficient anemia	1992, FDA	[160]
Doxil/Caelyx	Liposomes	Doxorubicin	Multiple myeloma	1995, FDA	[161,162]
DexFerrum/DexIron	Iron dextrane colloid		Iron deficient anemia	1996, FDA	[163]
Depocyt	Liposomes	Cytarabine	Lymphomatous meningitis	1999, FDA	[164,165]
FerrIecit	Iron gluconate colloid		Anemia treatment in patients with chronic kidney disease	1999, FDA	[166]
Venofer	Iron sucrose colloid		Anemia treatment in patients with chronic kidney disease	2000, FDA	[167]
Oncaspar	Liposomes	Asparaginase	Acute lymphoblastic leukemia	2006, FDA	[168]
Feraheme	Iron polyglucose sorbitol carboxymethylether colloid		Anemia treatment in patients with chronic kidney disease	2009, FDA	[169]
Monofer	Iron isomaltoside colloid		Iron deficient anemia when oral method do not work or iron delivery is required immediately	2009, some of Europe	[170]
Marqibo	Liposomes	Vincristine	Acute lymphoblastic leukemia	2012, FDA	[171,172]
Diafer	Iron isomaltoside colloid		Iron deficient anemia	2012, some of Europe	[173]
Injectafer/Ferinject	Iron carboxymaltose colloid		Iron deficient anemia	2013, FDA	[174]
Vyxeos	Liposomes	Daunorubicin and cytarabine	Acute myeloid leukemia	2017, FDA	[175–177]

**Table 2:** Drugs already approved by FDA for other application that are now studied or under clinical trials to for hematological malignancies applications

Name	Composition		Disease	Year-Target FDA approval	References
	NPs	Active substances			
DaunoXome	Liposomes	Daunorubicin	Acute Myeloid/Lymphoblastic Leukemia	1996-HIV Kaposi’s sarcoma	[178–180]
Myocet	Liposomes	Doxorubicin	Lymphoma	2000-metastatic breast cancer	[181,182]
Abraxane	Albumin	Paclitaxel	Lymphoma	2005-breast, lung and pancreatic cancer	[183]

**Table 3:** Drugs currently under clinical trials

Name	Composition		Disease	Clinical trial.gov identifier (Phase)	References
	NPs	Active substances			
ABI-011	Albumin	Thiocolchicine analog	Lymphoma	NCT02582827 (I) NCT01163071 (i)	[184]
AZD2811	Polymers	Aurora B kinase inhibitor	Acute myeloid leukemia	NCT03217838 (I, II)	[109,185]
BP1001	Liposomes	Growth factor receptor bound protein-2 antisense oligonucleotide	Leukemia	NCT02923986 (I, II) NCT02781883 (II) NCT01159028 (I)	[186–188]
DCR-MYC	Liposomes	DsiRNA for MYC oncogene silencing	Multiple myeloma and lymphoma	NCT02110563 (I)	[189,190]
JVRS-100	Liposomes	Plasmid DNA complex	Leukemia	NCT00860522 (I)	[191]
Mitoxantrone hydrochloride liposome	Liposomes	Mitoxantrone	Leukemias and lymphoma	NCT02043756 (I) NCT02131688 (I) NCT02856685 (I, II) NCT03776279 (II) NCT02595242 (I) NCT02597387 (II) NCT02597153 (II) NCT02879643 (I) NCT02518750 (II) NCT02733380 (II) NCT02724163 (III) NCT03591510 (II)	[192–194]
NC-4016 DACH-Platin micelle	Polyamino acid and PEG	Oxaliplatin	Lymphoma	NCT03168035 (I)	[195]
PNT2258	Liposomes	Single-stranded DNAi	Lymphoma	NCT02378038 (II) NCT02226965 (II) NCT01733238 (II) NCT01191775 (I)	[196–198]
